# Diagnosis of Tobacco Amblyopia

**Published:** 1880-02

**Authors:** 


### DIAGNOSIS OF TOBACCO AMBLYOPIA.

Due consideration has not been given to this subject in England, and Mr. Edward Nettleship, Ophthalmic Surgeon to St. Thomas' Hospital (Vol. 9, 1879), makes this contribution :

The symptoms in tobacco amblyopia all depend on the fact that this amblyopia, the central region of the visual field is the first part affected, and remains throughout, the seat of the greatest relative defect, and, further, that the periphery of the visual field remains of full size.

1. However much acuteness of central vision may be lowered, the patients show no difficulty or awkwardness in walking about, and have not the "amaurotic aspect" so common in the subjects of progressive atrophy of the nerves, in whom the peripheral contraction of the field is an early event, and may lead to difficulty in avoiding obstacles, and to the other inconveniences which arise from loss of indirect sight, before central vision is much affected.

2. The sight in tobacco amblyopia is nearly always best in a rather dull light, the complaint being very much the same as in early uncomplicated nuclear cataract. The patients when asked whether the sight is always bad alike, generally say that it is better the first thing in the morning and again towards evening, that it is better on dull days, and that in direct sunlight it is very bad.

3. The state of the visual field: In tobacco amblyopia the greatest functional defect is always found to occupy an oblong or oval patch, which extends from the fixing point (corresponding to the yellow spot) out towards and often beyond the blind spot (corresponding to the disc). This patch ("central scotoma" of authors) is only defective (amblyopic), never blind (amaurotic); it is a "relative," not an "absolute" scotoma. Indeed, it is so relative that patients do not often make complaints that would lead us to suspect its existence, whereas, in the absolute scotoma caused, *e. g.*, by a large hemorrhage at the yellow spot, the symptoms are usually very sugestive of the state. Still, in many tobacco cases we notice that the patient in attempting to read moves the print about as if trying to find some position in which it is better seen. This symptom is best seen in early and slight cases, when print of moderate size (say 10 J.) can still be read, and the patient, not having got accustomed to the defect, is constantly trying to see smaller print.

The scotoma of tobacco amblyopia is invariably symmetrical in the two eyes, indicating affection of parts of the retinæ or nerves which correspond anatomically, but are not physiologically identical—a fact which is strongly due to a change in the orbital part of the optic nerves, and not to any cerebral or central lesions; and it is conjectured that the changes may chiefly occur in the fibres at the periphery of the nerve, which are believed to supply the central part of the retinæ.

It is not pretended that accurate measurement of the field of vision is commonly necessary, except to emphasize the value of some of the more easily appreciable symptoms which have been mentioned, and which, although characteristic enough, may be overlooked and sought for. These are, besides the improvement of vision in dull sight, the history of equally symmetrical failure of sight without other ocular symptoms, and without cerebral or spinal symptoms, excepting often nervousness and tremulousness; the presence in many cases of some degree of color-blindness for green and red, and sometimes for yellow, much more noticeable if the colored objects be small spots, and frequently passing undetected if large masses of color be used for testing; the failure of vision, generally gradual, but occasionally rapid, or at any rate noticed suddenly; the pupils natural in size and activity, or at most rather sluggish and somewhat too large. If it be added that there are no signs of chronic glaucoma, nor of incipient cataract, that the ophthalmoscopic changes are very slight in comparison with the defect in sight, being limited in most cases to (1) congestion and (2) more or less pallor of the optic alone, and the spectacles suitable for patient's age and refraction do not bring sight up to the normal, we shall see that the diagnosis of this disease usually presents no difficulties, and that it might often indeed be made even without ophthalmoscopic examination.

Affections of sight capable of being easily confused with those which form the subject of this paper may be sought for in vain among the non-smokers. It is no doubt rare to see the disease in smokers who are water-drinkers, and very common to see it in those who have damaged their stomach and their nervous system by drink. But the more we study this disease, the stronger does the evidence seem that without tobacco this common characteristic form of amblyopia would be almost unknown. In respect to treatment, it is before all things essential to believe in the advice we give; general directions to "smoke less and drink less" are, as a rule, useless. Complete permanent abstinence must be insisted on, for though something short of this would, as many cases show, often be enough, it is quite unsafe to give any discretion to the patient in respect to so insidious a habit.—*N. C. Med. Journal.*

